# Risk factors for prolonged hospitalization and disease course in osteomyelitis: a single−centre cohort study

**DOI:** 10.3389/fcimb.2026.1654595

**Published:** 2026-08-19

**Authors:** Qiangsheng Feng, Yuejuan Song, Xiaoqin Ha

**Affiliations:** Department of Clinical Laboratory, The 940th Hospital of Joint Logistics Support Force of People’s Liberation Army, Lanzhou, China

**Keywords:** clinical characteristics, disease course, hospitalization days, osteomyelitis, pathogen

## Abstract

**Objective:**

To investigate pathogen epidemiology and clinical characteristics in osteomyelitis patients for optimizing clinical management strategies.

**Methods:**

Confirmed osteomyelitis cases required pathogen isolation with radiological correlation. The cohort included both confirmed (culture−positive) and probable (culture−negative but meeting clinical and radiological criteria) cases, Radiation-induced osteomyelitis, sclerosing osteomyelitis and drug−associated osteomyelitis. Clinical specimens underwent standardized culture and VITEK Compact II or MALDI-TOF MS identification. SPSS 22.0 analyzed data via Mann-Whitney U tests and logistic regression for prolonged outcomes.

**Results:**

Analysis of 704 osteomyelitis patients revealed a male predominance (2.5:1 ratio) with median age at presentation of 41.4 years (IQR 27.0–55.0). Mortality was low (0.7%), but disability/amputation occurred in 3.53%. Patients experienced median cumulative hospitalization of 22 days (IQR 15.0-38.2) and disease duration of 4.7 months (IQR 1.5-13.6), with 79.7% undergoing surgical intervention (primarily lesion resection [22.7%] or debridement [14.2%]). The tibia was the most frequent infection site (31.5%), followed by femur (18.4%) and Jawbone (9.7%). Fracture-related infections predominated pathogenetically (47.7%), showing tibial (47.3%) and femoral (23.8%) predominance with *Staphylococcus aureus* (50.4%) as the leading pathogen. Microbiological confirmation was achieved in 61.9% (439/704) of cases, yielding 546 isolates. *S.aureus* predominated (50.8%), with 35.4% being MRSA (all susceptible to vancomycin/linezolid/tigecycline). Fracture-related infection (OR 2.24, p<0.001), femur involvement (OR 3.09, p<0.001), tibia involvement (OR 2.53, p<0.001), pathogen-positive culture (OR 3.56, p<0.001), MRSA (OR 2.64, p<0.001), and polymicrobial infection (OR 2.62, p<0.001) as significant predictors of prolonged hospitalization (>30 days). For prolonged clinical disease course (>6weeks), significant predictors included fracture-related infection (OR 1.83, p<0.001), femur involvement (OR 1.93, p=0.003), pathogen-positive culture (OR 2.66, p<0.001), *S. aureus* isolation (OR 1.79, p=0.001).

**Conclusion:**

In conclusion, fracture, femur involvement, *S. aureus*, and positive pathogen culture predict prolonged osteomyelitis hospitalization and disease course, jaw and finger bone sites predict shorter hospitalization. Pathogen epidemiology and infection site dictate clinical management strategies.

## Highlights

- S. aureus (50.8%) is the predominant pathogen in osteomyelitis, with 35.4% of isolates being MRSA but fully susceptible to vancomycin/linezolid/tigecycline.
- Fracture−related infection, tibial involvement, S. aureus isolation, MRSA, and polymicrobial infection significantly predict prolonged hospitalization.
- Jawbone and finger bone involvement are associated with shorter hospitalization days.

## 1 Introduction

Osteomyelitis is a serious bone and joint infection caused by a wide range of pathogens, including bacteria and fungi (Lew and Waldvogel, 2004; Landman, 2010). It frequently develops as a complication of fracture co−infection, trauma, joint replacement, diabetic foot infection, soft tissue infections, or bacteremia (Schmitt, 2017). The metaphysis of long bones, particularly the tibia and femur, is particularly susceptible to osteomyelitis due to its rich but sluggish blood flow, which facilitates bacterial seeding and persistence (Berbari et al., 2015). Clinically, osteomyelitis may present with recurrent episodes, purulent discharge, and, in severe cases, sequestrum or cavity formation, potentially leading to lifelong disability or even life−threatening complications (Mader et al., 1999b). Accurate etiological diagnosis and appropriate antimicrobial therapy are critical, and microbiological culture combined with histopathological examination remains the gold standard (Conterno and Turchi, 2013).

Despite significant advances in diagnostic imaging and surgical techniques, the management of osteomyelitis remains challenging, largely due to its heterogeneous etiologies, diverse clinical presentations, and the growing threat of multidrug−resistant organisms (Tande and Patel, 2014; Senneville et al., 2024). Several epidemiological studies have described the pathogen distribution and risk factors for osteomyelitis in various settings (Kremers et al., 2015; Ren et al., 2023). However, most previous studies have been limited by small sample sizes, single−center designs, or a narrow focus on specific patient populations (e.g., diabetic foot or post−traumatic cases). Moreover, few large−scale studies have systematically analyzed the site−specific and pathogen−dependent risk factors for prolonged hospitalization and disease course in a comprehensive cohort of osteomyelitis patients. In particular, the interplay between infection source (e.g., hematogenous, contiguous spread, post−traumatic) and clinical outcomes remains incompletely understood, and data on the comparative impact of different surgical interventions on prognosis are scarce.

Given these gaps, the present study aims to provide a thorough analysis of the clinical characteristics, etiological spectrum, and treatment outcomes in a large cohort of 704 osteomyelitis patients treated at a tertiary hospital over a 12−year period. We specifically focus on identifying independent risk factors for prolonged hospitalization and prolonged disease course, with an emphasis on infection site, pathogen type, and patient comorbidities. By clarifying these associations, this study seeks to inform risk stratification, guide empirical antimicrobial therapy, and optimize surgical decision−making, thereby contributing meaningful evidence to the field of osteomyelitis management.

## 2 Materials and methods

### 2.1 Study design

**Figure 1:**
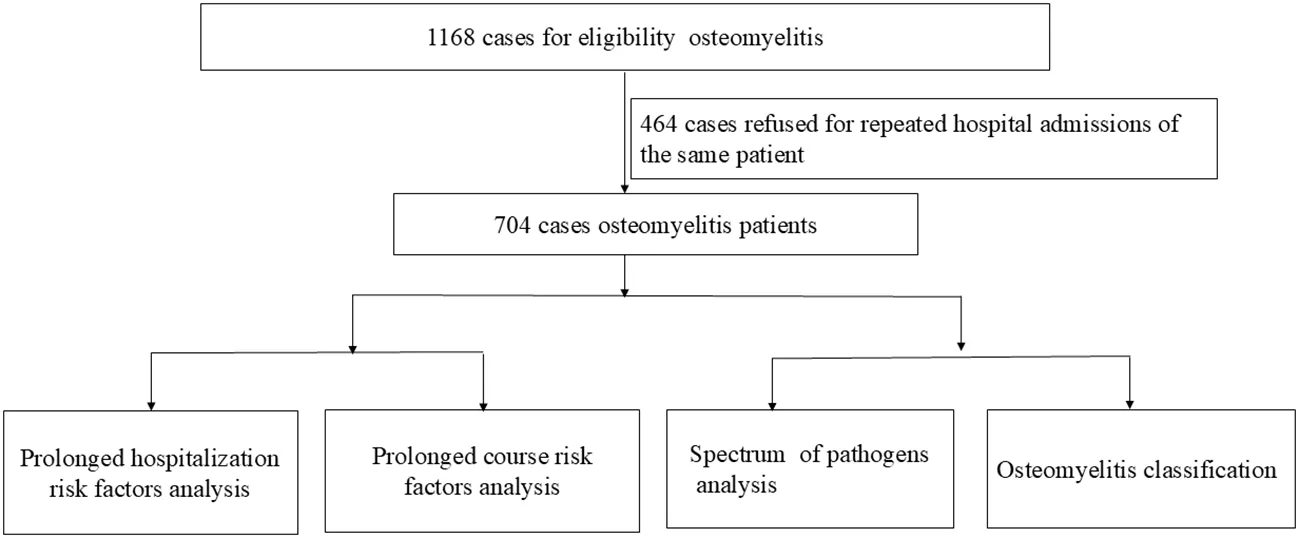
Selection criteria for the inclusion of patients with osteomyelitis. The Digital Medical Record System was queried for all patients admitted between January 2013 to January 2025 with a diagnosis of osteomyelitis, using a keyword searches for “osteomyelitis,”.

Selection criteria for the inclusion of patients with osteomyelitis(**Figure 1**) The Digital Medical Record System was queried for all patients admitted between January 2013 to January 2025 with a diagnosis of osteomyelitis, using a keyword searches for “osteomyelitis,” The initial search yielded 1,168 unique admission records. Patients with multiple hospital admissions for the same osteomyelitis episode were counted only once (i.e., the first admission was retained, and subsequent admissions for the same infection were excluded).

### 2.2 Inclusion and exclusion criteria

The diagnosis of osteomyelitis was established according to the criteria recommended by the European Federation of National Associations of Orthopaedics and Traumatology (EFORT) (Mijuskovic et al., 2018; McNally et al., 2020; Miller et al., 2024).

Confirmed osteomyelitis: Patients who met at least two of the following three criteria:

1. Clinical evidence: Localized pain, swelling, erythema, purulent discharge, or sinus tract formation, with or without systemic signs (fever, chills).
2. Radiological evidence: Characteristic imaging findings on MRI or CT, including bone marrow edema (T2−weighted fat−saturated MRI hyperintensity) or cortical destruction combined with periosteal reaction and adjacent soft−tissue abscess.
3. Microbiological evidence: In open fractures or implant−associated infections, isolation of the same pathogenic bacterium from at least two deep−tissue specimens (e.g., bone biopsy, intraoperative sample).

Probable osteomyelitis: Patients who met clinical and radiological criteria (criteria 1 and 2) without microbiological confirmation.

Radiation-induced osteomyelitis (14 cases) is defined as secondary infection occurring in irradiated, devitalized bone tissue. It is characterized by a prolonged clinical course, frequent recurrence, and limited response to conventional therapy, primarily due to compromised vascular supply and impaired tissue healing capacity.

Sclerosing osteomyelitis (5 cases) is a chronic, non−suppurative form of osteomyelitis characterized by prominent bone sclerosis rather than sequestrum formation or abscess. The classic subtype, Garré’s sclerosing osteomyelitis, commonly affects the jawbone or long bones, presenting with recurrent local pain and periosteal reaction.

Drug−associated osteomyelitis(4cases), most commonly presenting as medication−related osteonecrosis of the jaw (MRONJ), is a complication of therapy with antiresorptive (e.g., bisphosphonates, denosumab) or anti−angiogenic agents. It is defined by the presence of exposed necrotic bone in the maxillofacial region for >8 weeks, in the absence of prior radiation therapy.

### 2.3 Data source and sampling process

Using the Hospital Information System (HIS), we identified hospitalized patients with osteomyelitis between 2013 and 2025. For each patient’s first recorded clinical episode, we collected demographic data, medical history, surgical interventions, infected bone site(s), disease course, total hospitalization duration, and prognosis. Data collection complied with relevant guidelines and regulations.

### 2.4 Definitions

Debridement is defined as the medical removal of devitalized, necrotic, or contaminated tissue, foreign bodies, and microbial biofilm from a wound or infection site to facilitate healing and prevent the spread of infection. Techniques include surgical (sharp), enzymatic, autolytic, and biological debridement.

Prolonged accumulated hospitalization is defined as a total hospital stay exceeding 30 days. This clinically meaningful threshold was chosen based on established literature and clinical consensus (Mijuskovic et al., 2018). In sensitivity analyses, hospitalization was also analysed as a continuous variable (log−transformed) to confirm robustness.

Disease course is defined as the interval from the onset of clinical symptoms (e.g., local pain, swelling, fever) to clinical resolution or the end of active treatment (e.g., wound healing, normalization of inflammatory markers. Prolonged disease course is defined as >6 weeks (42 days), based on the EFORT classification of chronic osteomyelitis (Mijuskovic et al., 2018).

Polymicrobial infection defined as isolation of two or more distinct bacterial species from the same bone/tissue specimen within 48 hours.

Disability/amputation is defined as loss of limb or persistent functional impairment preventing return to pre−infection activities.

Mortality is defined as all−cause in−hospital mortality.

### 2.5 Covariates and adjustment strategy

Demographic and clinical characteristics were analyzed as exposure variables. Categorical variables (sex, age group, S. aureus infection, MRSA status, pathogen type, disease course category, bone infection site, and infection source.

### 2.6 Bacterial culture and antimicrobial susceptibility test

#### 2.6.1 General bacterial culture

**Figure 2:**
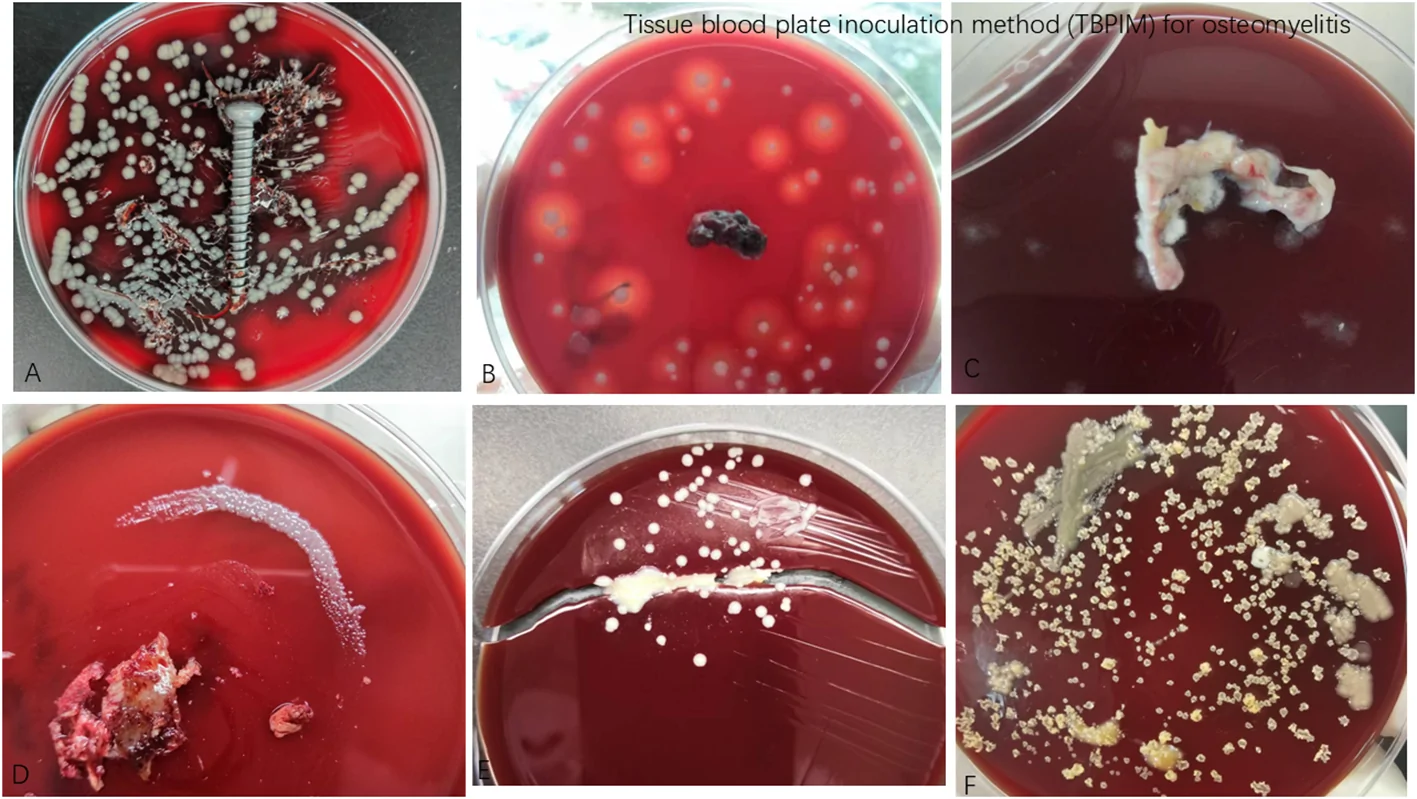
Tissue blood plate inoculation method (TBPIM) for osteomyelitis. **(A)** Fixed screw culture 24h, S.aureus. **(B)** Osteomyelitis tissue culture, S.aureus. **(C)** Osteomyelitis tissue culture, S. apiospermum. **(D)** Native Vertebral Osteomyelitis tissue culture 72h, B. melitensis. **(E)** Knee joint tissue culture 48h,Candida albicans. **(F)** Osteomyelitis patients Skin soft tissue culture72h: Nocardia otitidiscaviarum.

All specimens (bone tissue, aspirate fluid, pus, joint fluid, secretions, and wound exudates) were collected aseptically and transported to the clinical microbiology laboratory within 2 hours. Empirical antibiotic therapy was initiated immediately after obtaining deep cultures in patients with sepsis or severe local inflammation, based on local antimicrobial susceptibility patterns (e.g., vancomycin + piperacillin/tazobactam). In clinically stable patients, antibiotics were withheld until culture results (Berbari et al., 2015). Tissue and bone specimens obtained during clinical procedures were processed by selecting fragments of 2–6 mm³. These fragments were inoculated onto blood agar plates using the Tissue blood plate inoculation method (TBPIM) for osteomyelitis (**Figure 2**); To validate the sensitivity and specificity of TBPIM, we separately collected 30 fixed metal samples from patients with confirmed osteomyelitis. The culture method is shown in **Figure 2**. Pus, secretions, and sinus puncture fluid were directly inoculated onto blood agar plates. Puncture fluids and joint fluids were centrifuged at 3,000 rpm for 5 minutes; 50 µL of sediment was then inoculated onto blood agar plates.All inoculated plates were incubated at 35°C under 5% CO_2_ for 72 hours.Biological safety cabinets were used for all procedures. Anaerobic, fungal, and mycobacterial cultures were performed routinely for bone biopsy specimens and selectively for other specimens based on clinical indication.

Specimen hierarchy: Bone biopsy > deep tissue > sinus tract > pus > blood culture > implant samples. For non−sterile site specimens, contamination was distinguished from true infection by requiring two positive cultures with the same organism or clinical correlation with response to targeted therapy.

#### 2.6.2 Blood culture

For patients with suspected osteomyelitis, blood cultures were collected in paired BacT/ALERT^®^ (bioMérieux) and BACTEC™ (BD Diagnostics) bottles. All samples were incubated in automated systems for ≥7 days.When the instrument signaled a positive culture, Gram staining was immediately performed. Laboratory personnel reported preliminary results to the attending physician within 15 minutes of detection.For potential skin contaminants (e.g., coagulase-negative staphylococci or α-hemolytic streptococci), ≥2 identical organisms from separate blood draws were required to confirm clinical significance.

#### 2.6.3 Pathogen identification and antimicrobial susceptibility test

After the formation of bacteria, pathogen identification and antibiotic sensitivity testing were performed using GN, GP, GN16, and GP67 cards on the VITEK Compact II instrument or MALDI-TOF MS. Pathogen and antibiotic sensitivity test results are reported to the clinical department through the Laboratory Information System (LIS). Guidelines for drug sensitivity test refer to CLSIM100 (2024 edition) (Clinical and Laboratory Standards Institute, 2024).

### 2.7 Statistical methods, model specifications, and handling of confounders

Statistical analyses were performed using SPSS 22.0 (IBM Corp., Armonk, NY). Normality of the continuous variables (disease course and total hospitalization days) was assessed using the Shapiro−Wilk test; both variables showed significant deviation from a normal distribution (p < 0.001, n = 704). Therefore, continuous variables - disease course and total hospitalization days - were presented as median with interquartile range (IQR; P25, P75). For the binary outcomes, prolonged hospitalization was defined as >30 days, and prolonged disease course as >6 weeks (42 days). For univariate comparisons between two independent groups — including sex, age group, S. aureus infection, MRSA status, pathogen type, disease course category, positive pathogen culture, and surgical status — the Mann−Whitney U test was used to examine their associations with the continuous outcome variables (disease course and total hospitalization days).For comparisons involving more than two groups — such as infection site, infection source, surgical type, hospitalization count, and surgical count — the χ² test was applied.

**Figure 3:**
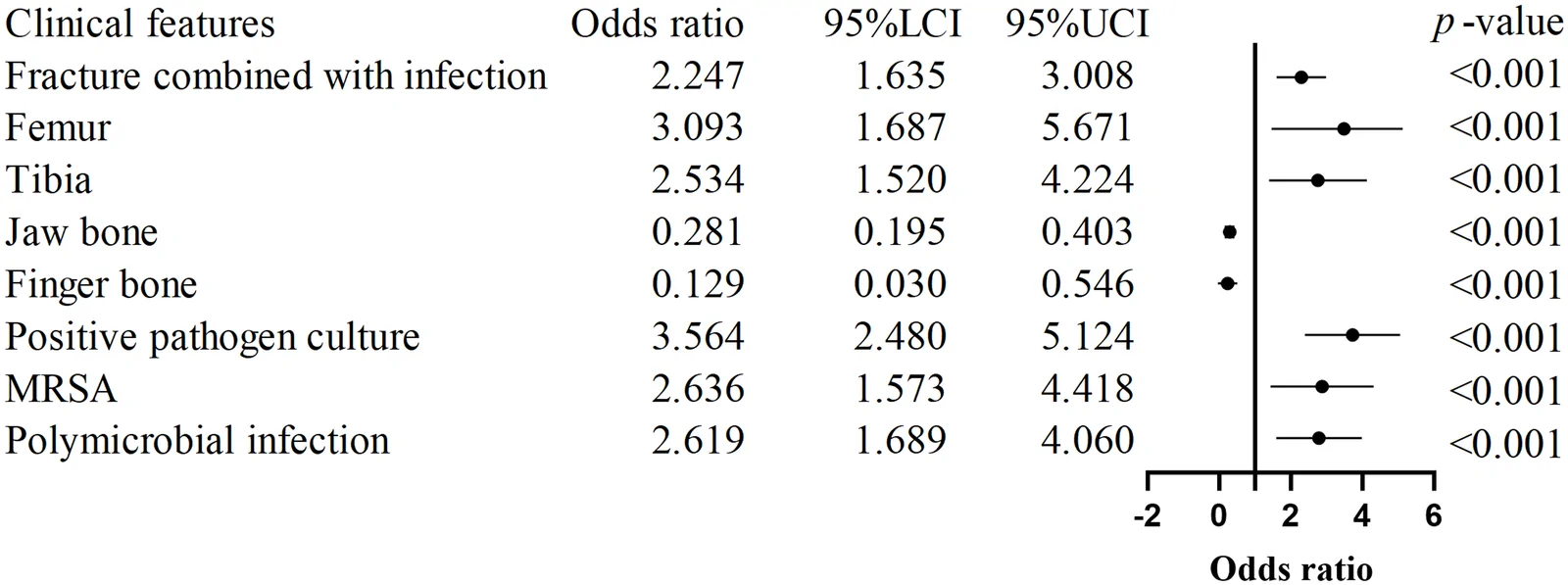
Factor prolonged hospitalization (>30 days) in osteomyelitis patients Mann-Whitney U test and logistic regression analysis shown that fracture combined with infection, infection bone, positive pathogen culture, co-pathogen, MRSA, and *S.aureus* was significant differences and *p-value <*0.001.

**Figure 4:**
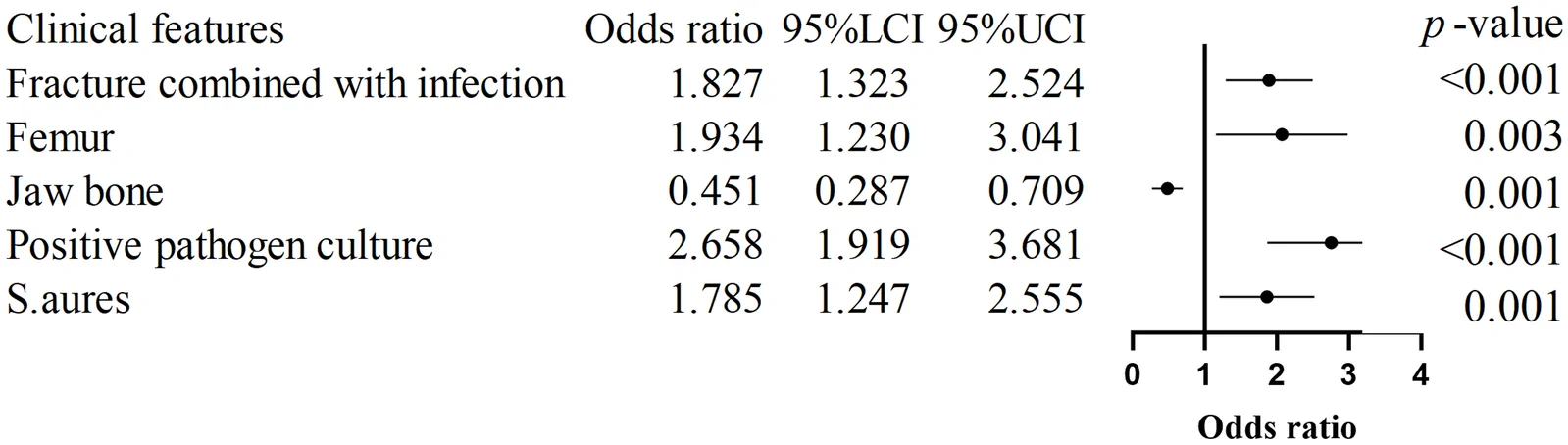
Factor for prolonged disease course (>6 weeks) in osteomyelitis patients Mann-Whitney U test and logistic regression shown that infection source, infection bone, and pathogen species showed significant differences and *p-value <*0.001.

Variables with p<0.01 in univariate analysis were entered into the multivariable logistic regression models, with additional clinically relevant variables (age, sex, diabetes) forced into the models. Complete−case analysis was used (missing data <5% for all variables). The events−per−variable ratio was 30 events per variable, which is adequate for the number of variables included. Interaction terms were examined between site, pathogen, and fracture−related infection; no significant interactions were found. Reference categories: for infection site, reference = “other sites” (all non−tibial/non−femoral sites); for pathogen, reference = “other pathogens” (non−S. aureus, non−MRSA, non−polymicrobial). The results of logistic regression (odds ratios, confidence intervals, p−values) are presented in **Figures 3**, **4** (forest plots). A sensitivity analysis using continuous outcome models (log−transformed linear regression) was performed to confirm the robustness of the findings; the results were consistent with the primary analysis. A sensitivity analysis restricted to the 439 culture−confirmed cases was also performed, and the results were consistent with the main analysis.

## 3 Results

### 3.1 Clinical characteristics and treatment and outcomes of patients with osteomyelitis

**Figure 5:**
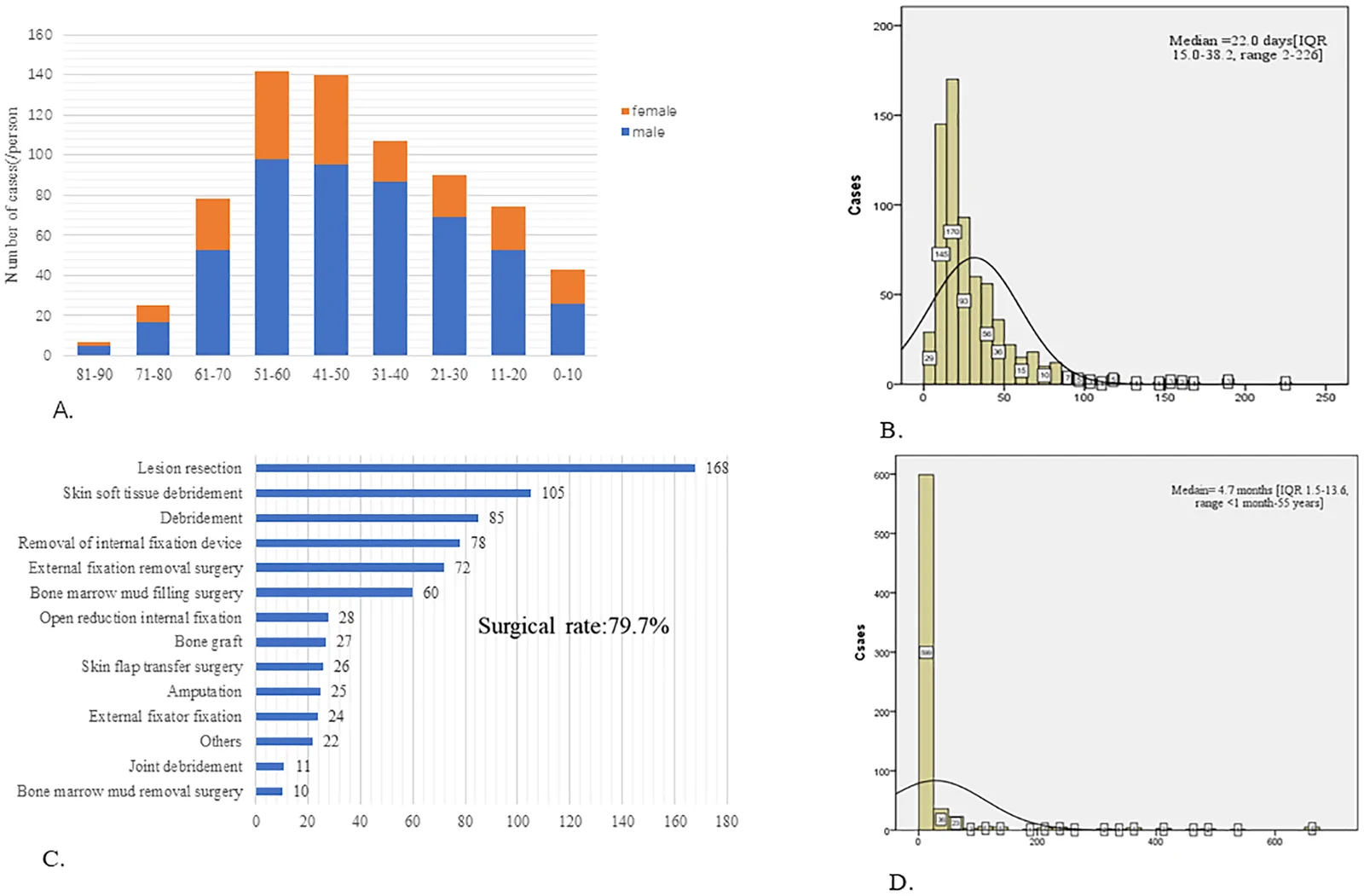
General clinical characteristics. ① The median age of the patients was 41.4(IQRrange 0-90 years), and the age 31-60 years accounted for 55.3%. ② The accumulated hospitalization days IQR was 22.0 (15.0,38.2) days/person (range2-226 days).③The surgical number IQR was 1.0(1.0,2.0) time/person (range 0-7 times) and surgical rate accounted for 79.7%, the main surgery was lesion resection and skin soft tissue debridement accounted for 22.7% and 14.2% respectively.④ The course IQR was 4.7 (1.0,13.6) months (range was <1month to 55years). (A) Age group (year), (B) Accumulated hospitalization days(d), (C) Surgical type (n=741,Time), (D) Course of disease (month,n=704 cases).

Among 704 osteomyelitis patients, mortality was 0.7% (n=5) and disability/amputation occurred in 3.53% (n=25). The cohort showed male predominance (ratio 2.5:1, 503:201) and non-normally distributed age at onset (range 0-90 years; median 41.4 years). Hospitalization metrics included: admission frequency median 1.0 [IQR 1.0-2.0, range 1-8]/person, total hospitalization days median 22.0 [IQR 15.0-38.2, range 2-226], and disease course median 4.7 months [IQR 1.5-13.6, range <1 month-55 years]. Surgical intervention occurred in 79.7% (n=561) with median 1.0 procedures/person [IQR 1.0-2.0, range 0-7]; primary procedures were lesion resection (22.7% of surgeries) and debridement (14.2%) (**Figures 5A–D**). All patients received antibiotic therapy: susceptibility-directed regimens when available, and empirical treatment otherwise.

### 3.2 Osteomyelitis: site specific distribution and etiological categories

**Figure 6:**
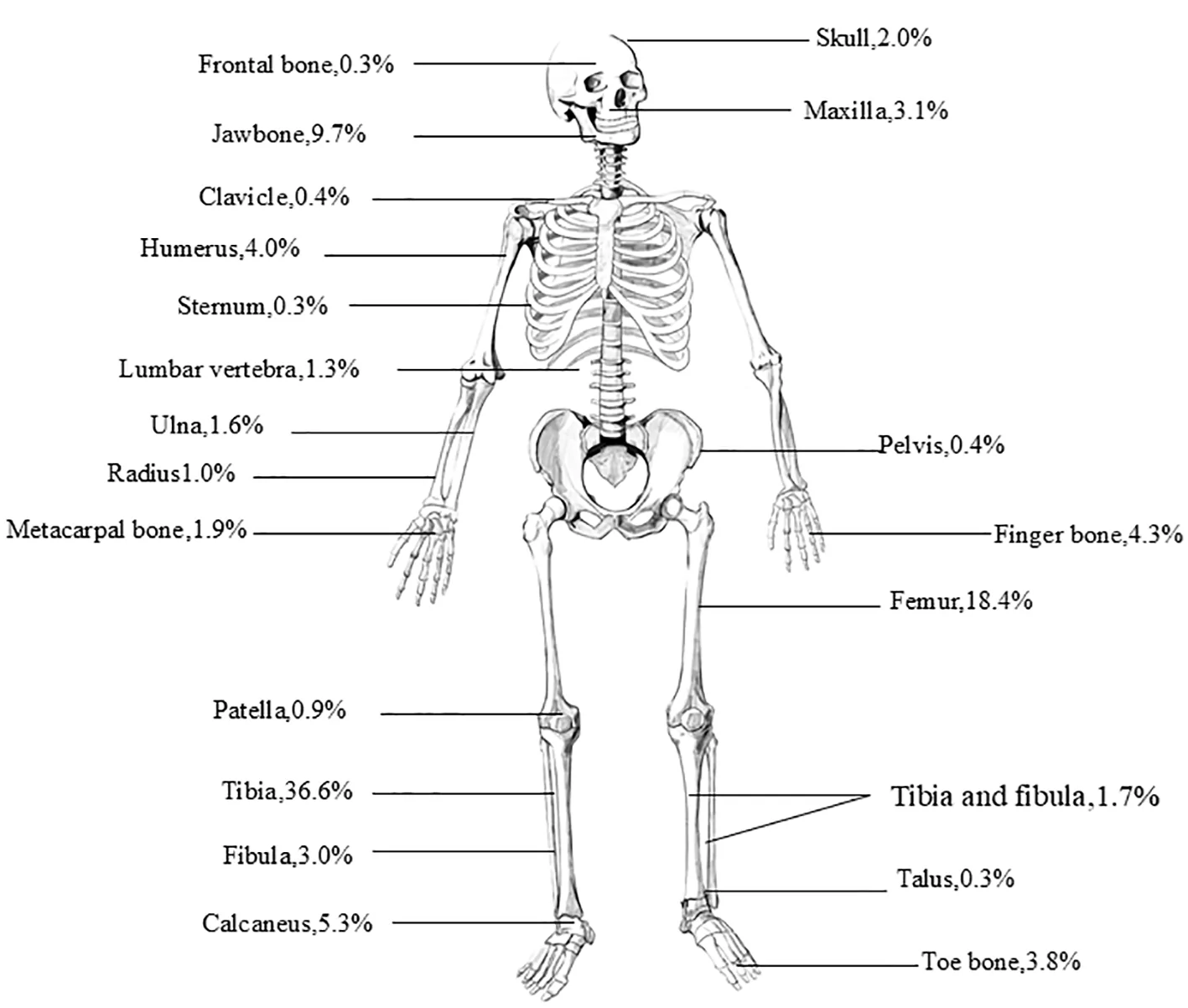
Pathogen detected and drug resistance rate for S. aureus in osteomyelitis. Pathogen including C+c, G-b, *Candida* spp.,and *TB* accounted for 41.2%,18.3%,0.9%,1.1%, respectively. *S. aureus* was the first pathogen accounting for 50.8% (223/439) and with co-pathogens were at 26.6%, MRSA rate at 35.4%,.

In a cohort of 704 osteomyelitis patients, the tibia was the most frequent infection site (31.5%), followed by the femur (18.4%), Jawbone (9.7%), calcaneus (5.3%), and phalanges (4.3%) (**Figure 6**). Among 704 cases of osteomyelitis, 681 cases (96.7%) were classified as infectious, 23(3.3%) cases were classified as non−infectious osteomyelitis, comprising 14 cases of radiation−induced osteomyelitis, 5 cases of sclerosing osteomyelitis, and 4 cases of drug−associated osteomyelitis, (**Table 1**). Pathogenetically, fracture-related infections predominated (47.7%, n=335), with primary infection sites being the tibia (47.3%), femur (23.8%), and calcaneus (4.8%), and predominant pathogens including S. aureus (50.4%), E. cloacae (10.5%), and E. coli (5.8%). Contiguous-focus osteomyelitis originating from skin/soft tissue infections (12.4%, n=87) primarily affected the tibia (18.4%), jawbone (14.9%), and calcaneus (11.5%), with pathogens dominated by S. aureus (50.0%), viridans streptococci (22.0%), and P. aeruginosa (4.0%). Major subtypes included chronic hematogenous osteomyelitis (8.9%, n=63) and trauma-associated bone infection (5.5%, n=39), which resulted from road traffic accidents, falls, and combat-related injuries; other etiologies comprised osteomyelitis of the jawbone (5.1%, n=36), acute hematogenous osteomyelitis (5.0%, n=35), septic arthritis with bone involvement (4.8%, n=34), periprosthetic joint infection(PJI) (3.0%, n=21), radiation-induced osteonecrosis with infection (2.0%, n=14), diabetic foot osteomyelitis (1.8%, n=13), and medication-related osteonecrosis of the jawbone (0.6%, n=4).

**Table 1:** Osteomyelitis: classification of disease and bone involvement (n=704 cases, %).

Osteomyelitis classification	Cases	%	Infection site			Pathogen		
Fracture-related infection of the bones	336	47.7	Tibia(47.3%)	Femur(23.8%)	Calcaneus(4.8%)	*Staphylococcus aureus*(50.4%)	*Enterobacter cloacae* (10.5%)	*Escherichia coli*(5.8%)
Contiguous-focus osteomyelitis	87	12.4	Tibia(18.4%)	Jawbone (14.9%)	Calcaneus(11.5%)	*Staphylococcus aureus* (50.0%)	*V.streptococci*(22.0%)	*Ps.aeruginosa*(4.0%)
Chronic osteomyelitis	63	8.9	Femur(38.1%)	Tibia(23.8%)	Calcaneus(14.3%)	*Staphylococcus aureus* (47.4%)	*Brucella melitensis* (13.2%)	*M.tuberculosis*(5.3%)
Trauma-associated bone infection	39	5.5	Tibia(48.7%)	Finger bone(28.2%)	Toe bone(10.3)	*Staphylococcus aureus* (36.8%)	*Streptococcus*(10.5%)	*Enterobacter cloacae* (10.5%)
Osteomyelitis of the Jawbone	36	5.1	Jawbone (75.0%)	Maxilla(25.0%)		*V.streptococci* (88.9%)	*Eikenella corrodens*(11.1%)	
Acute osteomyelitis	35	5.0	Tibia(52.3%)	Femur(22.9%)	Fibula(11.4)	*Staphylococcus aureus*(96.0%)	*Salmonella typhi*(4.0%)	
Septic arthritis	34	4.8	Tibia(47.1%)	Femur(26.5%)	Humerus(16.7%)	*Staphylococcus aureus* (53.8%)	*Candida* spp.(7.7%)	*Klebsiella pneumoniae* (7.7%)
Periprosthetic joint infection	21	3.0	Tibia(28.6%)	Skull(23.8%)	Femur(9.5%)	*Serratia marcescens*(25.0%)	*Staphylococcus aureus* (16.7%)	*Staphylococcus epidermidis* (16.7%)
Radiation-induced osteomyelitis	14	2.0	Jawbone (71.4%)	Maxilla(28.6%)				
Diabetic foot osteomyelitis	13	1.8	Tibia(23.1%)	Femur(15.4%)	Toe bone(15.4)	*Staphylococcus aureus* (53.8%)	*Klebsiella pneumoniae*(15.4%)	*Escherichia coli* (7.7)
Sclerosing Osteomyelitis	5	0.7	Tibia(40.0%)	Femur(40.0%)	humerus(20.0%)	*Staphylococcus aureus*		
Drug-associated osteomyelitis	4	0.6	Jawbone (100%)			*V.streptococci*		
Lumbar osteomyelitis	5	0.7	Lumbar			*Escherichia coli*	*Brucella melitensis*	
Cranial osteomyelitis	5	0.7	Skull			*Staphylococcus aureus*	*Klebsiella pneumoniae*	

The remaining 7 cases comprised 3 cases of post−amputation osteomyelitis, 2 cases following bone grafting, 1 case secondary to multiple myeloma, and 1 case following finger replantation.

### 3.3 Microbiological findings and *S. aureus* drug resistance

**Figure 7:**
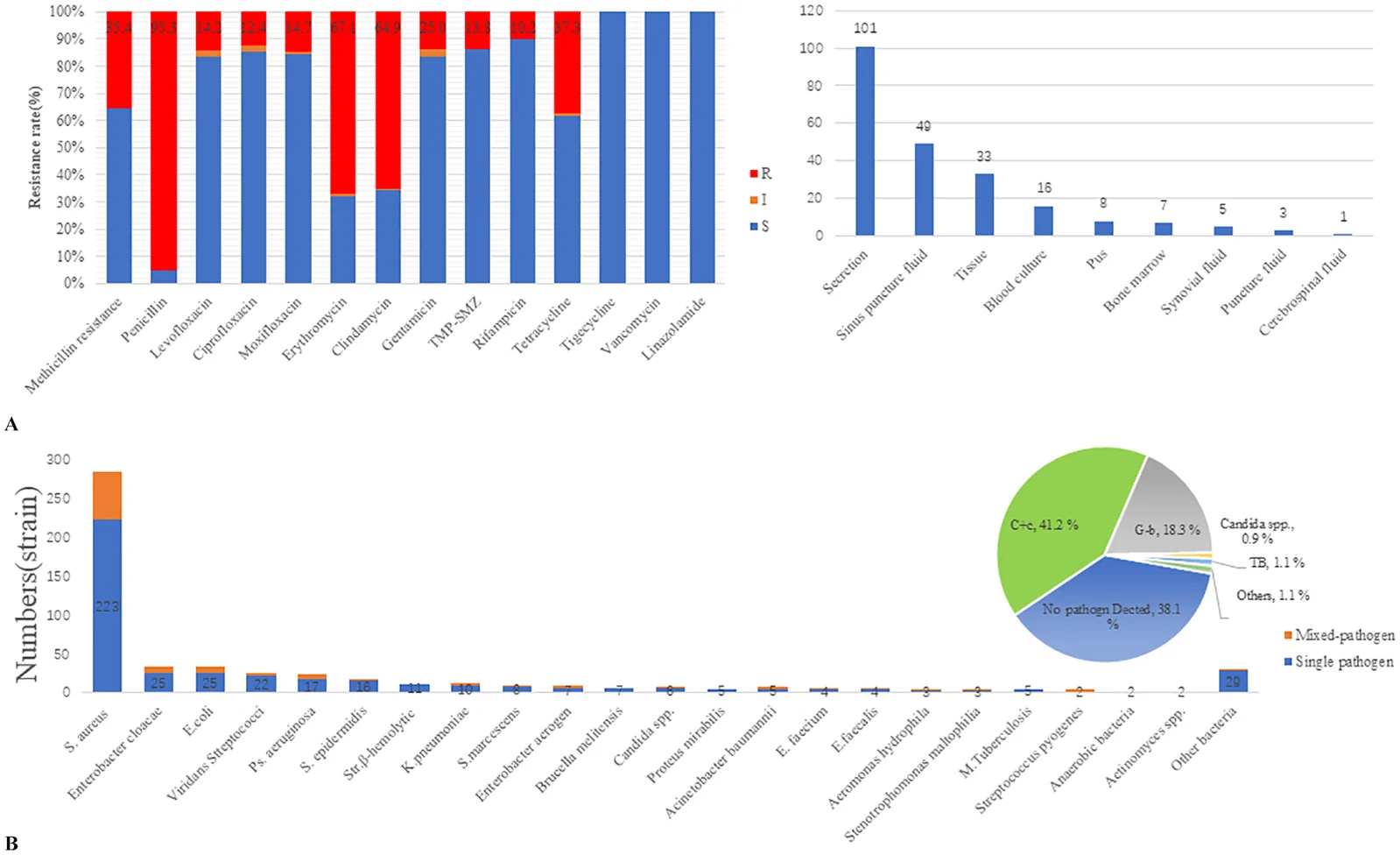
Infected bones and pathogenesis in osteomyelitis patients. The first suffered bone was tibia accounted for 31.5%, pathogenesis were mainly fracture with infection, chronic osteomyelitis. **(A)** Antimicrobial Resistance Profile and Specim en Sources of Staphylococcus aureus in Osteom yelitis Patients (n=233). **(B)** Spectrum and Frequency Distribution of Predominant Pathogens in Osteomyelitis Patients (n=439).

In a cohort of 704 osteomyelitis patients, 439 (61.9%) had microbiologically confirmed infections yielding 546 isolates from 68 species. Pathogen distribution comprised Gram-positive bacteria (41.2%), Gram-negative bacteria (18.3%), *Candida* spp. (0.9%), *Mycobacterium tuberculosis* (1.1%), culture-negative cases (38.1%), and polymicrobial infections (24.4%). *S. aureus* predominated (50.8%, 223/439), with polymicrobial involvement in 26.6% of S. aureus cases. Methicillin-resistant *S. aureus* (MRSA) prevalence was 35.4%, demonstrating universal susceptibility to tigecycline, vancomycin, and linezolid (**Figure 7A**). Other significant pathogens included *Enterobacter cloacae* (5.7%) and *Escherichia coli* (5.7% each), *Streptococcus viridans* group (5.0%), *Pseudomonas aeruginosa* (3.9%), β-hemolytic *streptococci* (3.6%), *Klebsiella pneumoniae* (2.5%), and *Serratia marcescens* (1.8%) (**Figure 7B**).

### 3.4 Factors analysis for accumulated hospitalization days and disease course

Mann-Whitney U tests and logistic regression analyses identified significant predictors for prolonged hospitalization days (> 30 days). Fracture-related infection (OR 2.247, 95%CI 1.635-3.008, p<0.001), femur involvement (OR 3.093, 95%CI 1.687-5.671, p<0.001), tibia involvement (OR 2.534, 95%CI 1.520-4.224, p<0.001), pathogen-positive culture (OR 3.564, 95%CI 2.480-5.124, p<0.001), MRSA (OR 2.636, 95%CI 1.573-4.418, p<0.001), and polymicrobial infection (OR 2.619, 95%CI 1.689-4.060, p<0.001) all showed significant associations (p<0.001, **Table 2**, **Figure 3**). Factor for Prolonged disease course (>6 weeks) in osteomyelitis patients, included fracture-related infection (OR 1.827, 95%CI 1.323-2.524, p<0.001), femur involvement (OR 1.934, 95%CI 1.230-3.041, p=0.003), pathogen-positive culture (OR 2.658, 95%CI 1.919-3.681, p<0.001), S. aureus isolation (OR 1.785, 95%CI 1.247-2.555, p=0.001) (**Table 2**, **Figure 4**).

**Table 2:** Factors for course and accumulated hospitalization days in osteomyelitis patients.

Factors	n (%)	Course IQR (P25, P75) (4.7 Month)	Z	*P*	Accumulated hospitalization 22 days(d) IQR (P25, P75)	Z	*P*
Sex			1.134	0.257		2.334	0.020
Male	503(71%)	5.0(1.0,12.0)			23.0(15.0,39.0)		
Female	201(29%)	4.0(1.0,12.0)			20.0(14.0,32.0)		
Age (year, mean=41.2year)			22.870	0.001		1.545	0.122
>=42	384(55%)	4.5(1.0,12.0)			21.0(14.0,37.0)		
<42	320(45%)	5.0(1.0,14.0)			23.0(16.0,40.0)		
*S.aureus*			0.476	0.634		2.167	0.030
Yes	224(51%)	6.0(2.0,17.0)			28.0(18.0,53.5)		
No	215(49%)	6.0(2.0,24.0)			28.0(16.0,43.0)		
MRSA			1.463	0.143		2.961	0.003
Yes	75(33%)	8.0(4.0,17.0)			39.0(22.0,68.0)		
No	149(67%)	6.0(1.0,20.5)			24.0(18.0,49.0)		
Pathogen			2.121	0.034		4.790	<0.001
co-pathogen	115(26%)	8.0(3.5,24.0)			39.5(21.0,70.0)		
single pathogen	321(74%)	6.0(2.0,15.5)			25.0(16.0,39.0)		
Positive pathogen culture			5.980	<0.001		9.026	<0.001
Yes	436(62%)	6.0(2.0,17.0)			28.0(17.0,48.0)		
No	269(38%)	2(1.0,12.0)			17.0(12.0,26.0)		
Duration of infection			3.024	0.002		2.212	0.027
Chronic osteomyelitis	63(63%)	12(2.0,114.0)			19.5(15.0,31.0)		
Acute osteomyelitis	35(37%)	2.5(1.0,10.0)			25.0(18.8,78.0)		
Infection site			χ2 = 34.660	<0.001		χ2 = 42.410	<0.001
Femur	129(26%)	12.0(2.0,36.0)			28.0(18.0,60.0)		
Tibia	270(55%)	6.0(2.0,12.0)			27.0(17.0,43.5)		
Jawbone	90(19%)	2.0(1.0,6.0)			16.0(12.0,26.0)		
Finger bone	30(7%)	2.0(1.0,3.0)			13.5(9.0,18.0)		
Surgical			4.333	<0.001		6.393	<0.001
Yes	560(80%)	6.0(1.0,15.0)			24.0(16.0,41.5)		
No	142(20%)	2.0(1.0,8.0)			16.0(11.0,27.5)		
Infection source			χ2 = 12.402	0.002		χ2 = 11.09	0.004
Fracture-related infection of the bones	336(74%)	6.0(2.0,17.0)			28.0(16.0,45.0)		
Soft tissue infections	87(19%)	3.0(1.0,12.0)			17.5(14.0,29.0)		
Septic knee	34(7%)	2.0(1.0,7.0)			25.0(20.0,42.0)		
Surgical type			χ2 = 8.459	0.015		χ2 = 3.149	0.369
Lesion resection	145(31%)	6.0(1.0,17.0)			23.0(14.0,39.0)		
Skin soft tissue debridement	153(33%)	4.0(1.0,12.0)			27.0(17.0,43.0)		
Fixation device	124(27%)	8.5(2.0,24.0)			27.5(16.0,50.5)		
Bone marrow mud filling surgery	44(9%)	5.0(2.0,18.5)			23.0(17.0,37.0)		
Surgical number(Time)			χ2 = 121.004	<0.001		χ2 = 142.825	<0.001
0	118(17%)	2.0(1.0,8.0)			16.0(11.0,28.0)		
1	386(55%)	2.0(1.0,12.0)			19.0(13.0,28.0)		
2	117(17%)	9.0(5.0,34.0)			25.5(26.0,48.5)		
3-7	81(11%)	17.0(9.0,36.0)			63.0(38.0,89.0)		
Hospitalization numbers (Time)			χ2 = 145.217	<0.001		χ2 = 235.355	<0.001
1	440(63%)	2.0(2.0,10.0)			17.0(12.0,23.0)		
2	164(23%)	7.0(4.0,15.5)			24.0(26.0,45.0)		
3-8	98(14%)	17.0(11.0,36.5)			61.5(39.0,89.0)		
Periods(year)			χ2 = 1.574	0.210		χ2 = 0.216	0.642
2013-2019	403(57%)	5.0(1.0,12.0)			30.7(14.0,38.0)		
2020-2025	301(43%)	4.5(1.0,17.0)			23.0(15.0,39.0)		

Data are median (IQR) or n (%). p values were calculated by Mann-Whitney U test, χ2 test.

The regression analysis was limited to infection sites with a sufficient number of cases (≥30 patients) to ensure statistical stability and meaningful odds ratio estimation. Infection site: reference = “other sites” (all non−tibial/non−femoral sites).Pathogen: reference = “other pathogens” (non−S. aureus, non−MRSA, non−polymicrobial). Model structure: Culture positivity, S. aureus, MRSA, and polymicrobial infection were not included in the same model. Instead, separate models were constructed to avoid nesting issues: Model 1: included culture positivity (yes/no) as a predictor. Model 2: included S. aureus (yes/no) and MRSA (yes/no) in separate models to avoid nesting. Model 3: included polymicrobial infection (yes/no).

To assess the robustness of our primary findings, we conducted sensitivity analyses using multiple linear regression models with natural log−transformed continuous outcomes (hospitalization days and disease course) to address their skewed distributions. For hospitalization days, fracture−related infection (standardized β = 0.167, p < 0.001), tibial involvement (β = 0.132, p < 0.001), femoral involvement (β = 0.177, p < 0.001), positive pathogen culture (β = 0.318, p < 0.001), polymicrobial infection (β = 0.211, p < 0.001), and MRSA infection (β = 0.173, p < 0.001) all remained significantly and positively associated with longer hospitalization. For disease course, fracture−related infection (β = 0.092, p = 0.014), femoral involvement (β = 0.177, p < 0.001), positive pathogen culture (β = 0.198, p < 0.001), polymicrobial infection (β = 0.211, p < 0.001), and S. aureus infection (β = 0.173, p < 0.001) remained significant positive predictors. These results were consistent in direction with the primary logistic regression analyses, indicating that our main findings are robust and not materially affected by the dichotomization of the outcome variables.

## 4 Discussion

Osteomyelitis, a destructive bone infection caused by microbial invasion, lacks comprehensive clinical classification and treatment guidelines (Masters et al., 2019). Our analysis reveals critical clinical burdens: a 3.53% amputation/disability rate, median cumulative hospitalization of 22 days, median disease course of 4.7 months, and 79.7% surgical intervention rate. These five deaths underscore comorbidities, septic shock, multidrug resistance, and financial barriers as key contributors to osteomyelitis mortality. These figures are comparable to those reported in a large European cohort, where the amputation rate for chronic osteomyelitis ranged from 4% to 8% and median hospitalization was 18–25 days (Conterno and Turchi, 2013). Chronic osteomyelitis is associated with prolonged disability and repeated hospitalizations, as shown in previous studies (Conterno and Turchi, 2013; Fantoni et al., 2019). In our cohort, 32.5% of patients reported persistent functional limitation at 12 months, but a formal quality−of−life assessment was beyond the scope of this retrospective study. A recent systematic review by Kremers et al. (2015) noted that up to 40% of patients with chronic osteomyelitis experience long−term functional impairment, which aligns with our findings (Kremers et al., 2015).

Anatomically, infections predominantly affected the tibia (31.5%), femur (18.4%), jawbone (9.7%), calcaneus (5.3%), and phalanges (4.3%), with fracture-related mechanisms driving tibial predominance (47.7% of fracture cases) - consistent with literature identifying open tibial fractures as the primary risk factor (Holtom and Smith, 1999; Mader et al., 1999a). Specifically, Gustilo−Anderson type III open fractures have been associated with post−traumatic osteomyelitis rates of 10−30%, and the tibia is the most commonly involved site (Gustilo and Anderson, 2002).

In our cohort, pathogen culture sensitivity for osteomyelitis was 61.9%, consistent with literature reporting 40% false-negative rates in microbiological testing (Kavanagh et al., 2018). Notably, to validate the experimental method of TBPIM, we cultured 30 fixed metal samples from patients with osteomyelitis who had been diagnosed with S. aureus osteomyelitis, with sensitivity and specificity of 100% – surpassing the 60.8% (periprosthetic tissue) and 78.5% (sonicate fluid) sensitivities reported by Trampuz et al. for prosthetic joint infections (Trampuz et al., 2007), and also exceeding the 69% culture positivity rate reported in a multicenter study of chronic osteomyelitis by Dudareva et al. (2018) (Dudareva et al., 2019). This warrants broader clinical adoption of this optimized method. However, the TBPIM validation was based on a small sample (n=30) and requires confirmation in larger prospective studies.

Among 546 isolates from 439 patients spanning 68 species, pathogen diversity encompassed Gram-positive cocci, Gram-negative bacilli, Candida spp., Mycobacterium tuberculosis, and polymicrobial infections (24.4%), underscoring the necessity of accurate etiological diagnosis for effective treatment (Landman, 2010; Hatzenbuehler and Pulling, 2011; Yeo and Ramachandran, 2014; Woods et al., 2021). S. aureus predominated (50.8%), with 26.6% involving co-pathogens. The MRSA prevalence (35.4%) aligns with U.S. data where S. aureus dominates acute hematogenous osteomyelitis and MRSA represents a major antimicrobial resistance concern (Funk and Copley, 2017; Urish and Cassat, 2020). However, a surveillance study reported a slightly lower MRSA prevalence (21%) in bone and joint infection, suggesting regional heterogeneity (Fu et al., 2023).

Secondary pathogens (constituting ~50% of non-S. aureus isolates) included Enterobacter cloacae, Escherichia coli, Streptococcus viridans group, Pseudomonas aeruginosa, β-hemolytic streptococci, *Klebsiella pneumoniae*, and *Serratia marcescens*. This profile corresponds with reported Gram-negative bacilli prevalence in bone biopsies (Zuluaga et al., 2002), given that certain pathogens (e.g., Gram−negative bacteria, Candida spp., Mycobacterium tuberculosis) are intrinsically resistant to vancomycin, empirical vancomycin monotherapy is inadequate, underscoring the continued necessity of culture−guided therapy. These risk factors are consistent with a large Chinese cohort study by Jiang et al. (2019), where fracture type and S. aureus infection independently predicted prolonged hospital stay (Berbari et al., 2015).

Multivariable analysis identified Fracture-related infection (OR 2.24, p<0.001), femur involvement (OR 3.09, p<0.001), tibia involvement (OR 2.53, p<0.001), pathogen-positive culture (OR 3.56, p<0.001), MRSA (OR 2.64, p<0.001), and polymicrobial infection (OR 2.62, p<0.001) as significant independent predictors prolonging cumulative hospitalization duration (>30 days). For disease course (>4 weeks), Factor for Prolonged disease course (>6 weeks) in osteomyelitis patients, included fracture-related infection (OR 1.83, p<0.001), femur involvement (OR 1.93, p=0.003), pathogen-positive culture (OR 2.66, p<0.001), S. aureus isolation (OR 1.79, p=0.001). To the best of our knowledge, these site−specific shorter disease course (jawbone and phalanges) has not been reported in the literature on osteomyelitis. Therefore, these site-specific associations and pathogen-dependent progression patterns are descriptive findings from our cohort; further studies with external validation are needed to confirm their generalizability. The association between positive culture and prolonged outcomes should be interpreted with caution, as culture positivity may correlate with severity, number of procedures, sampling quality, referral complexity, longer admission, or repeat admission. To address this, we performed a subgroup analysis restricted to the 439 microbiologically confirmed cases, which yielded results consistent with the main analysis. Separate models were constructed for culture positivity, S. aureus, MRSA, and polymicrobial infection to avoid nesting issues (MRSA is nested within S. aureus).

In summary, this study demonstrates the high clinical burden of osteomyelitis, identifies fracture, tibial location, positive pathogen culture, MRSA, and S. aureus as key predictors of prolonged hospitalization, and underscores the need for external validation of novel site−specific findings.

## 5 Limitations

This study has several limitations. First, its retrospective single−centre design may limit generalizability and introduce selection bias. Second, despite multivariable adjustment, residual confounding by unmeasured factors (e.g., SOFA/APACHE II scores, timing of source control) cannot be excluded. Third, the case definition included both confirmed and probable cases, which may introduce heterogeneity. Fourth, the dichotomization of continuous outcomes (hospitalization days and disease course), although clinically justified, may have reduced statistical power. Fifth, the TBPIM validation study used a small sample (n=30) and requires further validation in larger cohorts. Sixth, the study period spanned 12 years, during which diagnostic and treatment practices may have changed; however, a sensitivity analysis adjusting for calendar period (2013−2019 vs. 2020−2025) showed no significant period effect. Seventh, the absence of a structured follow−up protocol and lack of quality−of−life data are additional limitations. Eighth, the culture−negative cases (38.1%) could have been misclassified, and the low incidence of some pathogen subgroups may have underpowered certain analyses.

## 6 Conclusions

In conclusion, this study demonstrates the high clinical burden of osteomyelitis in a large Chinese cohort. Fracture−related infection, tibial involvement, S. aureus, MRSA, polymicrobial infection, and positive culture are key predictors of prolonged hospitalization. Jaw bone and finger bone sites are associated with shorter disease course. These findings underscore the importance of early source control, appropriate empirical antibiotic therapy, and vigilant management of high−risk patients. Future multicentre prospective studies are needed to validate these findings and to develop risk stratification tools for osteomyelitis management.

## Data Availability

The raw data supporting the conclusions of this article will be made available by the authors, without undue reservation.
